# Purification and Characterization of Flavonoids from the Leaves of *Zanthoxylum bungeanum* and Correlation between Their Structure and Antioxidant Activity

**DOI:** 10.1371/journal.pone.0105725

**Published:** 2014-08-26

**Authors:** Yujuan Zhang, Dongmei Wang, Lina Yang, Dan Zhou, Jingfang Zhang

**Affiliations:** College of Forestry, Northwest A & F University, Yangling, China; Islamic Azad University-Mashhad Branch, Mashhad, Iran, Islamic Republic of Iran

## Abstract

Nine flavonoids were isolated and characterized from the leaves of *Zanthoxylum bungeanum*. Their structures were elucidated by spectroscopic techniques as quercetin (**1**), afzelin (**2**), quercitrin (**3**), trifolin (**4**), quercetin-3-O-β-D-glucoside (**5**), isorhamnetin 3-O-α-L-rhamnoside (**6**), hyperoside (**7**), vitexin (**8**) and rutin (**9**). All compounds were isolated from the leaves of *Z. bungeanum* for the first time. Five compounds (**2, 4, 5, 6** and **8**) were found for the first time in the genus *Zanthoxylum*. To learn the mechanisms underlying its health benefits, *in vitro* (DPPH, ABTS, FRAP and lipid peroxidation inhibition assays) and *in vivo* (protective effect on *Escherichia coli* under peroxide stress) antioxidant activities of the nine flavonoids were measured. Quercetin and quercetin glycosides (compounds **1**, **3**, **5**, **7**, **9**) showed the highest antioxidant activity. Structure-activity relationships indicated that the -OH in 4′ position on the B ring and the -OH in 7 position on the A ring possessed high antioxidant activity; B ring and/or A ring with adjacent -OH groups could greatly increase their antioxidant ability. Also, due to the different structures of various flavonoids, they will certainly exhibit different antioxidant capacity when the reactions occur in solution or in oil-in-water emulsion. These findings suggest that *Z. bungeanum* leaves may have health benefits when consumed. It could become a useful supplement for pharmaceutical products and functional food ingredients in both nutraceutical and food industries as a potential source of natural antioxidants.

## Introduction

The genus *Zanthoxylum* (Rutaceae) consists of 250 species in the world, of which there are 45 species and 13 varieties in China. *Zanthoxylum bungeanum*, also called Sichuan pepper, is a common Chinese pepper, growing widely in the area of Sichuan, Shanxi, Shandong and Hebei Provinces of China. Just like other species in this genus, *Z. bungeanum* has a distinctive tingling taste. Due to its fresh aroma and taste, the dried fruits are used as one of the eight cuisine condiments and seasonings in China [1]–[2]. Apart from its common application as a condiment to make foods more flavoring, each part of *Z. bungeanum* have numerous medicinal virtues. In Traditional Chinese Medicine, the pericarp can be used for gastralgia and dyspepsia; the seed is reported to be antiphlogistic and diuretic; the leaves are considered carminative, stimulant and sudorific; the root can cure epigastric pains and treat bruises, eczema and snake-bites [3]–[7]. Owing to its high medicinal value, intensive investigation leads to isolate many classes of secondary metabolites such as flavonoids, alkaloids, amides, lignans, coumarins [8]–[11]. Thirteen flavonoids and ten unsaturated alkylamides were isolated from the pericarps of *Z. bungeanum* [9]–[12]; six alkaloids were isolated from the root of *Z. bungeanum* [13]. Phytochemicals, such as hydroxy-β-sanshool, xanthoxylin, hyperoside were found to stimulate the spontaneous beating rate (BR) of myocardial cells in culture [14]. These previous literatures indicated that *Z. bungeanum* can be used as pharmacological active products for improving human health in nutraceutical and pharmaceutical industries.

The young leaves of *Z. bungeanum* have been consumed as foodstuffs, and the mature leaves were used as condiments in traditional Chinese cusine. In some rural areas, local people eat the new leaves in spring seasons [15]. However, only a few people pay attention to the chemical work on the leaves of *Z. bungeanum*. Deng and coworkers found that the leaves of *Z. bungeanum* contained mainly nutritious and nutritional ingredients [15]. Yang and Xu provided preliminary data confirming that the leaves contain abundant flavonoids with good radical scavenging abilities [16]–[17]. These previous studies revealed that *Z. bungeanum* leaves are rich in flavonoids but studies on the isolation, purification and structure elucidation of individual polyphenols in the leaves of *Z. bungeanum* have not been reported yet. Since flavonols supplements in dietary food may evoke protective effects under peroxide stress, our current study was carried out to isolate the bioactive components present in the leaves of *Z. bungeanum*, in an attempt to gain a deeper understanding of the correlation between diet, health benefits and reduced risk of diseases. Furthermore, the authors attempted to evaluate the comparative antioxidant activity of the isolated compounds using several *in vitro* (DPPH, ABTS, FRAP and lipid peroxidation inhibition assays) and *in vivo* (protective effect on *Escherichia coli* under peroxide stress) methods. Also, the correlation between the structures of compounds and antioxidant capacity was discussed.

## Material and Methods

### General

Electrospray ion trap mass spectrometry (ESI-MS) was carried out with a Bruker ESI-TRAP Esquire 6000 plus mass spectrometry instrument. Nuclear magnetic resonance spectra (NMR) were recorded on a Bruker Avance III 500 MHz instrument in DMSO-*d6* using tetramethylsilane (TMS) as the internal standard. Analytical thin-layer chromatography (TLC) was performed with silica gel plates using silica gel 60 GF_254_ (Qingdao Haiyang Chemical Co., Ltd.). Sephadex LH-20 was purchased from GE Healthcare Bio-science AB (Sweden).

### Chemicals and reagents

1,1-Diphenyl-2-picrylhydrazyl (DPPH), 2,4,6-Tripyridyl-s-triazine (TPTZ), 2,2-azino-bis (3-ethyl-benzothiazoline-6-sulphonic acid) diammonium salt (ABTS), 6-hydroxy-2,5,7,8- tetramethylchroman-2-carboxylic acid (Trolox), (Sigma-Aldrich Co., St. Louis, USA); Thiobarbituric acid (TBA) (Guangdong Guanghua Chemical Factory Co., Ltd. PR China); Yeast extract, tryptone (Oxoid Ltd., Basingstoke, Hampshire, England). Deionized water (18 MΩ cm) was used to prepare aqueous solutions. All the chemicals used, including the solvents, were of analytical grade.

### Plant materials

*Z. bungeanum* leaves were collected from Taibai Mountains of Shaanxi province, in September, 2012, and authenticated by the Herbarium of the Northwest A&F University.

### Ethics statement

Specific permissions were not required for the described field sampling studies or for the collection of plant materials. For any locations/activities, no specific permissions were required. All locations where the plants were collected were not privately owned or protected in any way and the field studies did not involve endangered or protected species.

### Extraction, fractionation, and isolation

The air-dried and powdered leaves of *Z. bungeanum* (9.40 Kg) were extracted with 95% ethanol (1∶5 w/v) at room temperature (6 days×6), and the total filtrate was then concentrated by rotary evaporation under vacuum to obtain the ethanol extracts (1824.4 g). This extracts was further fractioned by column chromatography on silica gel (200–300 mesh, 120*10 cm), successively eluting with petroleum ether, chloroform, ethyl acetate, acetone and methanol. The eluents of the five different polarity solvents were collected separately and concentrated by rotary evaporation under vacuum to obtain five fractions (PEF, 105.9 g; CF, 112.7 g; EAF, 28.0 g; AF, 100.0 g and MF, 624.3 g). The EAF and AF fractions were screened as the most effective fractions. Thus, later purification and isolation were focused on EAF and AF.

The EAF (28.0 g) was initially chromatographed over a silica gel column (80*8 cm) using CHCl_3_ and MeOH under gradient conditions (9∶1→4∶1→1∶1→1∶4→0∶1) to yield 7 sub-fractions (E01–E07). Fraction 2 (E02, 8.6 g) was subjected to column chromatography over a silica gel column (60*4 cm) with a solvent mixture of CHCl_3_ and MeOH (30∶1→15∶1→9∶1→4∶1→1∶1) to yield compounds **1** (150.0 mg) and **2** (180.00 mg), respectively. Fraction 3 (E03, 10.6 g) was purified by silica gel with CHCl_3_:MeOH (20∶1→9∶1→4∶1→1∶1) to produce compound **3** (4.0 g). Fraction 4 (E04, 1.0 g) was subjected to column chromatography over a silica gel column (30*2) with a solvent mixture of CHCl_3_ and MeOH (15∶1→9∶1→4∶1→1∶1) and later a Sephadex LH-20 column (150*1 cm) with 100% MeOH to yield compound **8** (20.0 mg).

The AF (100.0 g) was chromatographed over a silica gel column (100*8 cm), using a mixed solvent of CHCl_3_ and MeOH (9∶1→4∶1→1∶1→1∶4→0∶1) to afford 5 fractions (A01–A05). Fraction 3 (A03, 5.0 g) was chromatographed over silica gel column (50*3) with a solvent mixture of CHCl_3_ and MeOH (30∶1→15∶1→9∶1→4∶1→1∶1) and Sephadex LH-20 column (150*1 cm) with 100% MeOH to yield compounds **5** (38.0 mg) and **6** (40.0 mg). Fraction 5 (A05, 20.0 g) was chromatographed over silica gel column (60*4 cm) with a solvent mixture of CHCl_3_ and MeOH (9∶1→4∶1→1∶1→1∶4) to afford 5 fractions (A0501–A0505). Fraction 2 (A0502, 300.0 mg) was chromatographed over silica gel with CHCl_3_ and MeOH (4∶1), followed by a Sephadex LH20 column (150*1 cm) with 100% MeOH to obtain compound **4** (40.0 mg). Fraction 3 (A0504, 5.8 g) was chromatographed over silica gel with a solvent mixture of CHCl_3_ and MeOH (15∶1→9∶1→4∶1→1∶1) to yield compound **7** (2.0 g) and **9** (45.0 mg).

**Figure 1:**
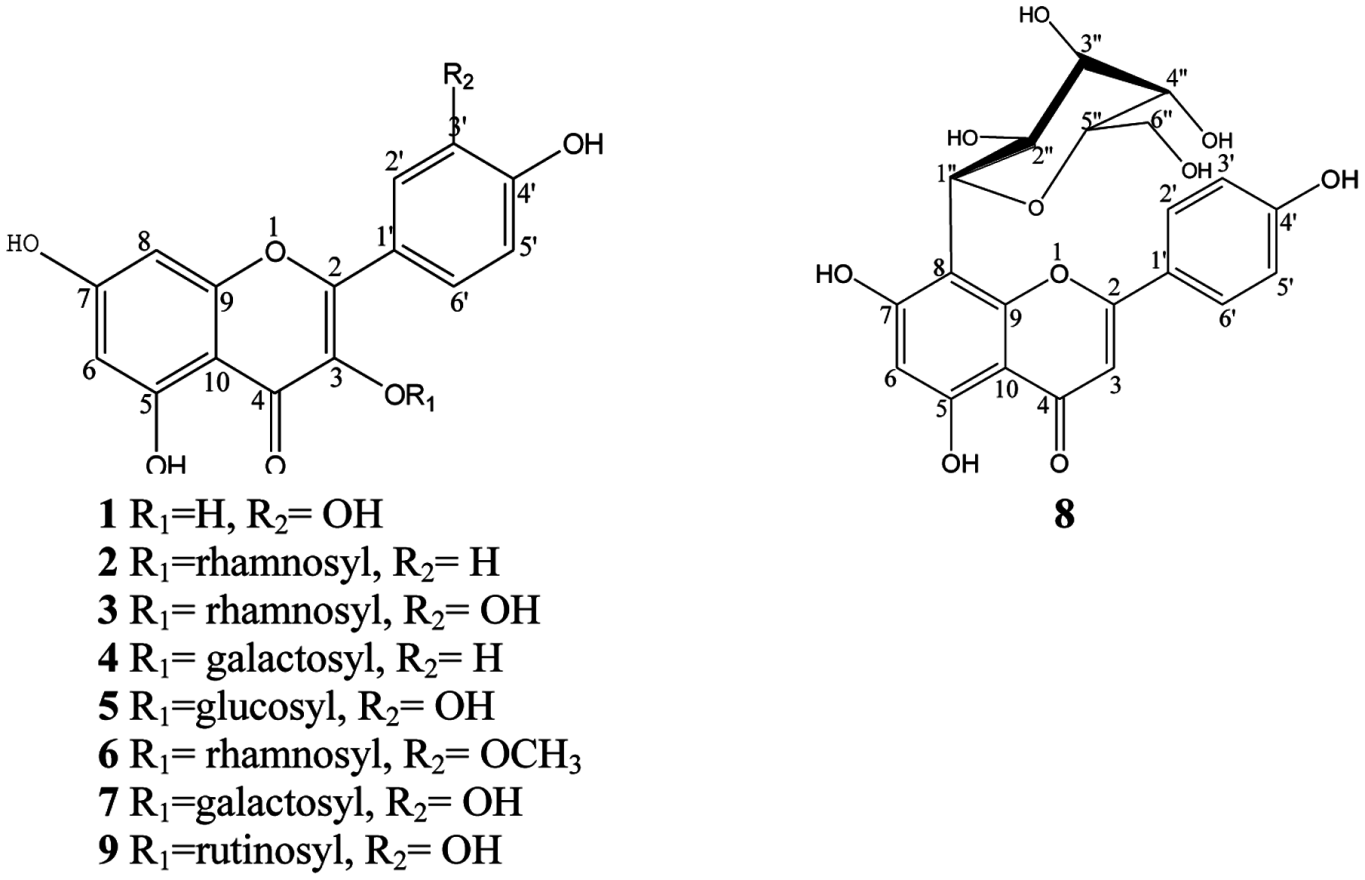
Structures of compounds 1–9.

**Figure 2:**
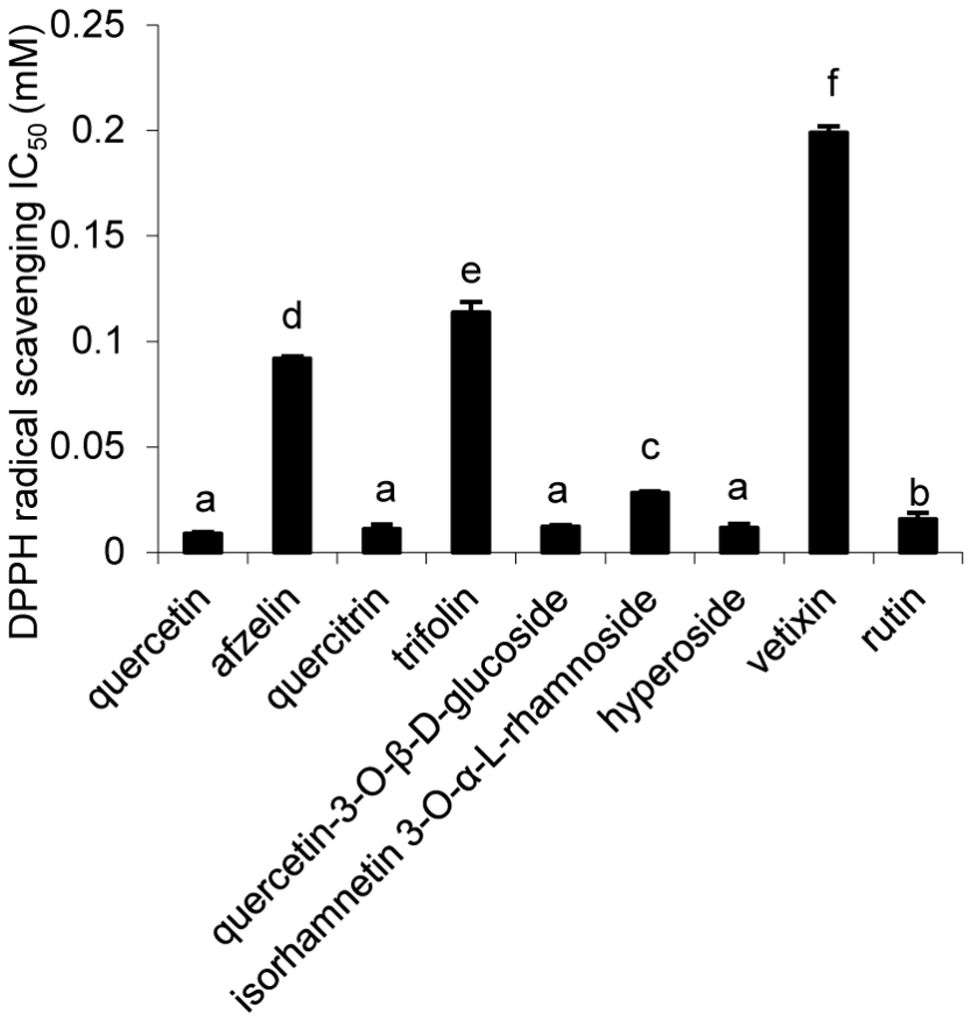
IC_50_ values of isolated compounds for DPPH radical scavenging activity (mean ± SD, n = 3). Bars with no letters in common are significantly different (P<0.05).

**Figure 3:**
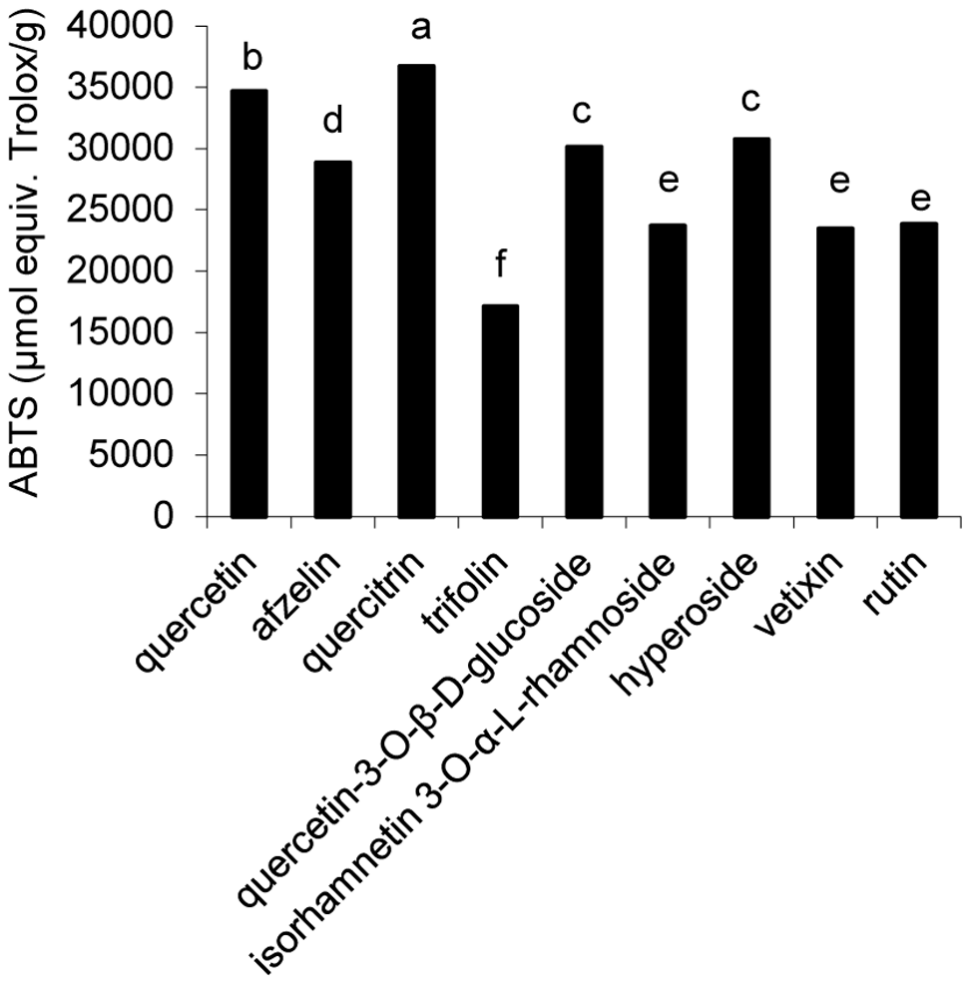
ABTS·^+^ radical scavenging activity of isolated compounds (mean ± SD, N = 3). Bars with no letters in common are significantly different (P<0.05).

All the isolated compounds **1–9** were characterized and identified by spectroscopic methods, as well as through comparison with published data. The spectral data were as follows and the structures are shown in Figure 1–3.

### Acid Hydrolysis of isolated compounds

Compounds **2–7**, and **9** (5.0 mg) was dissolved in 5.0 mL of 2 N HCl, heated at 90°C for 2 h, and then partitioned between ethyl acetate and water. Aglycon was recovered from the ethyl acetate layer and identified by direct comparison with an authentic sample. The water layers were identified by comparison with an authentic sample by TLC analysis. Sugars liberated from compounds **2**, **3** and **6** were identified as rhamnose; sugars liberated from compounds **4** and **7** were identified as galactose; sugars liberated from compounds **5** were identified as glucose; finally sugars liberated from compounds **9** were identified as glucose and rhamnose [18].

### DPPH radical scavenging assay

DPPH radical-scavenging activity was evaluated using the method described by Yen and Chen [19] with the following modifications [20]: a 2 mL volume of the sample solutions (0.003–0.2 mM) were added to 2 mL of DPPH solution (0.1 mM); and the absorbance was measured with a spectrophotometer at 517 nm 30 min later. All the tests and the controls were performed in triplicate. The DPPH free radical-scavenging activity was calculated using the following equation:
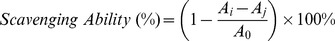



Where A_o_ is the absorbance of ethanol (2 mL) and DPPH· (2 mL), A_i_ is the absorbance of the tested sample (2 mL sample and 2 mL DPPH·), and A_j_ is the absorbance of the blank (2 mL sample and 2 mL ethanol). IC_50_ values were the effective concentrations at which DPPH radicals were scavenged by 50%, and were obtained from linear regression analysis.

### ABTS·^+^ radical cation decolorization assay

Antioxidant activity was determined using the decolorizing free radical ABTS·^+^ method as described previously [21]–[23]. An ABTS radical cation (ABTS·^+^) was produced by reacting an ABTS solution (7 mM) with 2.45 mM potassium persulfate, and the mixture was allowed to stand for 12–16 h in the dark at room temperature prior to use. The ABTS solution was diluted with PBS (pH 7.4) to the concentration that provided an absorbance value of 0.70 (±0.02) at 734 nm. For each analysis, 100 µL of samples (0.1 mM) were added to 3.9 mL of the ABTS·^+^ solution, and the decrease in absorbance at 734 nm was recorded within 6 min. The results were expressed as micromoles of trolox equivalent per gram dry weight of samples (µmol equiv. Trolox/g).

### Ferric reducing antioxidant power (FRAP) assay

The FRAP assay was performed with the following modifications [23]–[24]: the FRAP reagent was prepared daily by mixing 10 mL of a solution of ferric trichloride hexahydrate (20 mM), 10 mL of a solution of TPTZ (10 mM in 40 mM of hydrochloric acid) and 100 mL of 0.3 M acetate buffer (pH 3.6), and incubating them at 37°C. For each analysis, 400 µL of the samples (0.1 mM) were added to 3 mL of the FRAP solution. The increase in absorbance at 593 nm was recorded in 15 s intervals over the course of 30 min at 37°C. The FRAP results were expressed in terms of micromoles trolox equivalent per gram dry weight of samples (µmol equiv. Trolox/g).

### Lipid peroxidation inhibition assay

In this assay, a modified thiobarbituric acid reactive species (TBARS) assay was used to measure the lipid peroxide formed using egg yolk homogenates as lipid-rich media [25]–[27]. Briefly, fresh egg yolk emulsion was diluted to 10% v/v with 1.15% w/v KCl. 50 µL egg yolk emulsion, 50 µL of sample solution in different concentrations (0.003–0.2 mM), 150 µL of 20% (aqueous) trichloroacetic acid and 150 µL of 0.67% w/v thiobarbituric acid were added respectively and mixed thoroughly as the reaction solution. The whole reaction solution was then vortexed thoroughly and followed by incubation at 95°C in water bath for 1 h. After cooling, centrifuged the solution at 3000 rpm for 10 min. Absorbance of the upper layer was measured at 532 nm and percentage inhibition was calculated with the following formula:
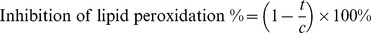
where c is the absorbance of fully peroxidized control and t is the absorbance of test sample. The IC_50_ value was calculated from the regression equation between sample concentration and rate of inhibition.

### Protective effect of isolated compounds on *Escherichia coli* growth under peroxide stress

*Escherichia coli* (ATCC No. 25922) was used to determine the antioxidant activity based on the method described by Smirnova with some modification [28]–[29]. Bacteria were grown overnight on Luria-Bertani (LB) medium at 37°C in 150 mL flask with shaking (50 r/min). Cell growth was monitored by measurement of the optical density at 600 nm. The mid-log phase bacteria (OD_600_ = 0.6) was diluted by fresh LB medium (final OD_600_ = 0.25±0.004). Then 1 mL of the cell solution were added to test tubes containing 8.9 mL of the LB medium and 100 µL of samples (0.1 mM) and incubated at 37°C with shaking (180 r/min). OD_600_ before cell addition was measured and subtracted for precise determination of growth. In 90 min when OD_600_ reached a value exactly equal to 0.342, bacteria were treated with hydrogen peroxide (6.5 mM) and incubated at 37°C with shaking (180 r/min) for 30 min. The absorbance was measured, and the growth was monitored for another 2–3 h. Specific growth rate was calculated according to the equation:
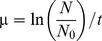
where μ is the specific growth rate and N_0_ and N are the optical density at time zero and t, respectively. The relative protective effect of compounds in this test was calculated as follows: t = 30 min, the specific growth rate of *E. coli* in medium containing compounds and 6.5 mM H_2_O_2_/the specific growth rate in medium containing only H_2_O_2_ (the control).

### Statistical analysis

All experiments were performed in triplicate and analyses of all samples were run in triplicate and averaged. Statistical analyses were performed with SPSS 18.0 for windows. The results were expressed as the means ± standard deviation (SD) of triplicate. The data were subjected to one-way analysis of variance (ANOVA) and the significance of difference between samples means was calculated by Duncan's multiple range test (SPSS Inc. Chicago).

## Results

### Antioxidant activities of the extracts and different fractions from *Z. bungeanum* leaves

Chromatographic fractionation of crude ethanol extracts led to five different polarity fractions (PEF, CF, EAF, AF, and MF fraction). The DPPH, ABTS radical scavenging activities and reducing power of individual fractions were then evaluated. As shown in Table 1, the EAF and AF fractions were screened as the most effective fractions with the highest DPPH radical scavenging abilities. Achieving a DPPH radical-scavenging activity of 50% required an EAF concentration of 13.20 µg/mL (IC_50_), which was not significantly different from that of AF (18.55 µg/mL), but was 3.1-fold lower than that of ethanol extract (p<0.05). Concerning the ABTS radical scavenging activity, we also found that EAF exhibited the highest activity (2147.83 µmol equiv Trolox/g), followed by AF (2044.58 µmol equiv. Trolox/g, p<0.05). The analysis of the results from the FRAP assay showed the same tendency when compared with the results of the scavenging capacity on DPPH and ABTS radical. It is exhibited that the reducing ability of EAF was 615.88 µmol equiv. Trolox/g, which was not significantly different from that of AF (594.15 µmol equiv. Trolox/g, p<0.05) but was 1.9-fold higher (p<0.05) than that of ethanol extracts. As a result, powerful antioxidants originating from *Z. bungeanum* leaves might mainly exist in two active fractions (EAF and AF). Thus, later purification and isolation were focused on EAF and AF.

**Table 1:** Antioxidant activities of the extracts and different fractions from *Z. bungeanum* leaves.

samples	DPPH IC_50_ (µg/mL)	FRAP (µmol equiv. Trolox/g)	ABTS (µmol equiv. Trolox/g)
ECE	40.75±0.21^b^	317.11±9.71^b^	1122.91±34.62^b^
PEF	377.95±39.39^e^	81.56±7.41^d^	264.20±37.27^e^
CF	169.15±4.60^d^	170.44±10.45^c^	563.86±22.66^d^
EAF	13.20±0.85^a^	615.88±1.86^a^	2147.83±23.08^a^
AF	18.55±0.35^a^	594.15±8.89^a^	2044.58±19.99^a^
MF	85.85±2.19^c^	191.93±2.22^c^	747.69±38.77^c^

ECE: ethanol crude extracts; PEF: petroleum ether fraction; CF: chloroform fraction; EAF: ethyl acetate fraction; AF: acetone fraction; MF: methanol fraction.

Each values represented in tables are means ± SD (N = 3).

Values with different letters (a, b, c, d, e) within same column were significantly different (P<0.05).

### Structure elucidation of isolated compounds

Nine compounds were identified from the leaves of *Z. bungeanum*. The individual structures of compounds **1–9** are shown in Figure 1.

Compound **1** was obtained as an amorphous yellow powder with the molecular formula C_15_H_10_O_7_, which was deduced from the ESI-MS *m*/*z*: 301.29 [M-H]^−^. The ^1^H and ^13^C NMR spectroscopic data indicated that **1** has a flavonol skeleton with 15 carbons, including five aromatic CH; ten quaternary carbons (one carbonyl, five O-bearing, and four aliphatic), suggesting that it is 3,5,7,3′,4′-pentahydroxyflavone, commonly known as quercetin [30]–[31]. Compound **3** was isolated as an amorphous yellow powder with the molecular formula C_21_H_20_O_11_, ESI-MS *m*/*z*: 447.77 [M-H]^−^. Further comparison of the ^1^H NMR and ^13^C NMR spectroscopic data of compounds **3**, **5**, **7** and **9** with those of compound **1** revealed that they all showed a typical flavonol pattern with a quercetin aglycon (Table 2). Compound **3** was determined as quercetin 3-O-α-L-rhamnoside (quercitrin), which was in accordance with the reported data [30], [32]–[33]. Compound **5** gives the molecular formula C_21_H_20_O_12_, ESI-MS m/z: 462.98 [M-H]^−^. Compared with the published literature, compound **5** was finally determined as quercetin 3-O-β-D-glucoside [33]. Compound **7** was obtained as an amorphous yellow powder. Its molecular formula was established as C_21_H_20_O_12_, ESI-MS m/z: 465.01 [M+H]^+^. Compared with the known literature, the structure of compound **7** was determined as quercetin 3-O-β-D-galactoside (hyperoside) [30], [33]. Compound **9** was isolated as an amorphous light yellow powder with the molecular formula C_27_H_30_O_16_, ESI-MS *m*/*z*: 609.91 [M-H]^−^. Through comparison with reported spectral data, compound **9** was identified as rutin [30]–[31].

**Table 2:** ^1^H, ^13^C, HMBC and COSY NMR spectroscopic data (500 MHz, DMSO-d_6_) of compounds 1, 3, 5, 7 and 9.

Position	Compound 1		Compound 3				Compound 5		Compound 7				Compound 9	
	δ^1^H	δ^13^C	δ^1^H	δ^13^C	HMBC	COSY	δ^1^H	δ^13^C	δ^1^H	δ^13^C	HMBC	COSY	δ^1^H	δ^13^C
2		147.28		156.93				156.78		156.79				156.91
3		136.14		134.60				133.76		133.99				133.81
4		176.27		178.23				177.90		177.97				178.01
5	12.47 br.s (6.9)	161.10	12.66 s	161.78	C5, C6, C10		12.65 s	161.70	12.63 s	161.71	C5, C6, C10		12.59 s	161.70
6	6.19 d (2.1)	98.65	6.21 br s	99.17	C8, C10, C5, C7	H8	6.21 d (2.0)	99.11	6.21 d (1.9)	99.16	C8, C10, C5, C7	H8	6.19 d (2.0)	99.17
7	10.93 s	164.30		164.69			10.88 s	164.59		164.66				164.69
8	6.42 d (2.1)	93.85	6.40 br s	94.10	C6, C10, C9, C7	H6	6.41 d (2.0)	93.96	6.41 d (1.9)	93.98	C6, C10, C9, C7	H6	6.38 d (2.0)	94.10
9		156.60		157.76				156.62		156.79				157.05
10		103.45		104.56				104.43		104.39				104.42
1′		122.40		121.23				121.62		121.59				121.66
2′	7.66 d (2.1)	115.47	7.30 d (1.5)	115.94	C6′, C4′, C2		7.53 d (J = 2.0)	115.66	7.53 d (2.0)	115.67	C6′, C4′, C2		7.53 d (2.1)	115.70
3′	9.35 s	145.49		145.68			9.23 s	145.27		145.30				145.23
4′	9.69 s	148.13		148.91			9.73 s	148.92		148.95				148.91
5′	6.88 d (8.4)	116.05	6.87 d (8.4)	116.15	C1′, C3′	H6′	6.87 d (9.0)	116.66	6.81 d (8.5)	116.44	C1′, C3′	H6′	6.84 d (9)	116.75
6′	7.53dd(8.4,2.1)	120.51	7.25 d (8.4)	121.59	C2′, C4′, C2	H5′	7.59 dd (9.0, 2.0)	122.06	7.67dd (8.5,2.0)	122.46	C2′, C4′	H5′	7.55 d (9, 2.1)	122.06
1″			5.27 s	102.33	C3, C2″	H2″	5.48 d (7.2)	101.30	5.38 d (7.8)	102.33	C3	H2″	5.35 d (7.4)	101.21
2″			3.99 dd	70.54		H1″, H3″		74.55	3.57 dd	71.70				74.57
3″			3.53 dd	71.05		H2″, H4″		76.95	3.39 dd	73.69				76.98
4″			3.17 dd	71.69		H3″		70.39	3.71 dd	68.41				70.51
5″			3.23 dd	70.86		H6″		78.04	3.33 dd	76.32				76.41
6″			0.82 d (6.0)	17.96		H5″		61.43	3.51 dd	60.62				67.47
1″′													5.12 d (1.9)	101.71
2″′														70.85
3″′														71.07
4″′														72.36
5″′														68.70
6″′													1.00 d (6.1)	18.18

Assignments were done by 1D (^1^H, ^13^C, DEPT) and 2D (COSY, HSQC, HMBC) NMR experiments.

Compound **2** was also obtained as an amorphous light yellow powder with the molecular formula C_21_H_20_O_10_, ESI-MS *m*/*z*: 431.80 [M-H]^−^. The^1^H NMR and ^13^C NMR (Table 3) spectra of compound **2** and compound **4** were compared with the data of known compounds and showed a typical flavonol pattern with a kaempferol aglycon. Compared with the known literature, the structure of compound **2** was determined as kaempferol 3-O-α-L-rhamnoside (afzelin) [30], [34]–[35]. Compound **4** gives the molecular formula C_21_H_20_O_11_, ESI-MS m/z: 447.20 [M-H]^−^. Compared with the known literature, compound **4** was finally determined as kaempferol 3-O-β-D-galactoside (trifolin) [30].

**Table 3:** ^1^H, ^13^C, HMBC and COSY NMR spectroscopic data (500 MHz, DMSO-d_6_) of compounds 2, 4, 6 and 8.

Position	Compound 2				Compound 4		Compound 6			Compound 8		
	δ^1^H	δ^13^C	HMBC	COSY	δ^1^H	δ^13^C	δ^1^H	δ^13^C	COSY	δ^1^H	δ^13^C	COSY
2		156.97				156.83		156.90			164.41	
3		134.70				133.71		134.67		6.78 s	102.94	
4		178.20				178.01		178.21			182.54	
5	12.63 s	161.77	C5, C6, C10		12.62 s	161.69	12.66 s	161.76		13.17 s	156.48	
6	6.21 d (1.6)	99.19	C8, C10, C5,C7	H8	6.21 d (1.9)	99.17	6.21 br s	99.14	H8	6.27 s	98.67	
7	10.86 s	164.68				164.63		164.66		10.83 s	162.91	
8	6.42 d (1.6)	94.21	C6, C10, C9,C7	H6	6.44 d (1.9)	94.14	6.40 br s	94.08	H6	6.78 s	105.26	
9		157.70				156.85		157.76			160.89	
10		104.62				104.43		104.54			104.55	
1′		121.00				121.34		121.18			122.11	
2′	7.77 dd (8.58)	131.06	C6′, C2, C4′	H3′	8.07 d (8.8)	131.45	7.30 d (1.5)	115.91		8.02 d (8.7)	129.39	H3′
3′	6.93 dd (8.58)	115.87	C5′, C1′, C4′	H2′	6.87 d (8.8)	115.53		148.90		6.91 d (8.7)	116.32	H2′
4′	10.2 s	160.46				160.43		145.67		10.35 s	161.61	
5′	6.93 dd (5.58)	115.87	C3′, C1′, C4′	H6′	6.87 d (8.8)	115.53	6.88 d (8.4)	116.10	H6′	6.91 d (8.7)	116.32	H6′
6′	7.77 dd (8.58)	131.06	C2′, C2, C4′	H5′	8.07 d (8.8)	131.45	7.25 d (8.4)	121.57	H5′	8.02 d (8.7)	129.39	H5′
3′- OCH_3_							3.19 s	49.06				
1″	5.31 d (1.8)	102.27	C3, C2″	H2″	5.40 d (7.5)	102.14	5.26 s	102.29	H2″	4.70 d (9.8)	73.88	H2″
2″	3.99 dd (3.6, 1.5)	70.55		H1″, H3″		71.68	3.98 dd	70.51	H1″, H3″	3.85 dd	71.39	
3″	3.48 dd	71.08		H2″, H4″		73.57	3.50 dd	71.05	H2″, H4″	3.29 dd	79.18	
4″	3.15 dd	71.61		H3″		68.35	3.17 dd	71.63	H3″	3.52 dd	71.09	
5″	3.17 dd	70.83		H6″		76.25	3.23 dd	70.80	H6″	3.26 dd	82.29	
6″	0.81 d (6.09)	17.93		H5″		60.67	0.82 d (6.0)	17.96	H5″	3.76 dd	61.82	

Assignments were done by 1D (^1^H, ^13^C, DEPT) and 2D (COSY, HSQC, HMBC) NMR experiments.

Compound **6** was obtained as an amorphous yellow powder with the molecular formula, C_22_H_22_O_11_, ESI-MS at *m*/*z*: 462.79 [M-H]^−^. ^13^C NMR spectrum showed signals for one -OCH_3_ (Table 3). The ^1^H NMR and ^13^C NMR (Table 3) spectra of compound **6** were compared with the data of known compounds and showed a typical flavonol pattern with an isorhamnetin aglycon. Acid hydrolysis of compound **6** gave L-rhamnose, identified by comparision with an authentic sample. The sugar portion was examined by TLC analysis. The ^1^H-NMR spectrum of compound **6** displayed two doublets at δ 5.26 (1H, d, *J* = 7.0 Hz) for the anomeric protons. Based on the coupling constant *J* = 1.15 Hz lesser than 4.0 Hz, the sugar configurations could be identified as α-L-rhamnose, which correlated with signals at 102.29 in the HSQC spectrum. In the HMBC (Figure 2), the anomeric proton signal of the rhamnose at δ 5.26 (H-1″) correlated with the carbon signal at δ 134.67 (C-3). These indicated that the rhamnose was attached to C-3 of the aglycone. Based on the above evidences and detailed analyses of the NMR spectra, the structure of compound **6** was determined as isorhamnetin 3-O-α-L-rhamnoside [30], [36].

Compound **8** was isolated as an amorphous light yellow powder with the molecular formula C_21_H_20_O_10_, ESI-MS *m*/*z*: 431.28 [M-H]^−^. The ^1^H NMR and ^13^C NMR (Table 3) spectroscopic data of compound **8** revealed that compound **8** bears similar flavonoid skeleton as compound **2** and the same sugar moieties as compound **5**. Yet a remarkable upfield shift (Δδ 11.05) for the C-8 of compound **8** (δ 105.26) compared to compound **2** (δ 94.21), and a remarkable downfield shift (Δδ 31.76) for the C-3 of compound **8** (δ 102.94) compared to compound **2** (δ 134.70), indicated that the sugar moiety was attached to C-8 instead of C-3, which was in accordance with the reported NMR spectroscopic data of vitexin [30], [37]–[38]. Unambiguous assignments of the ^1^H and ^13^C NMR data and the relative configuration were deduced from the COSY and HMBC experiments.

To the best of the author's knowledge, this is the first report of flavonoids **1–9** isolated from the leaves of *Z. bungeanum*. Compounds **2, 4, 5, 6** and **8** were found for the first time in the genus *Zanthoxylum* [3], [8]–[11]. Furthermore, the 2D-NMR data of the five known compounds **2, 3, 6, 7, 8** were given in this paper.

### DPPH radical scavenging activity of isolated compounds

quercetin and quercetin glycosides (quercetin, quercitrin, quercetin-3-O-β-D-glucoside, hyperoside and rutin) ranked with the most potent antioxidant activity. In particular, quercetin, quercitrin, quercetin-3-O-β-D-glucoside and hyperoside exerted predominant DPPH· radical scavenging activity with respective IC_50_ values of 0.009±0.001, 0.011±0.001, 0.012±0.001 and 0.011±0.001 mM, followed by rutin and isorhamnetin 3-O-α-L-rhamnoside with IC_50_ values of 0.016±0.001 and 0.028±0.001 mM, respectively (p<0.05). Kaempferol glycosides (afzelin and trifolin) were also found to possess significant DPPH· radical scavenging activity, while the C-glycoside flavonol (vitexin) exhibited poor DPPH· radical scavenging activity (Figure 2).

### ABTS·^+^ radical cation decolorization of isolated compounds

All the isolated compounds exhibited potent ABTS·^+^ scavenging activity (>17000 µmol equiv. Trolox/g), though quercetin (**1**), quercitrin (**3**), quercetin-3-O-β-D-glucoside (**5**) and hyperoside (**7**) ranked with the most potent ones (>30000 µmol equiv. Trolox/g) (Figure 3).

### Ferric reducing antioxidant power (FRAP assay) of isolated compounds

**Figure 4:**
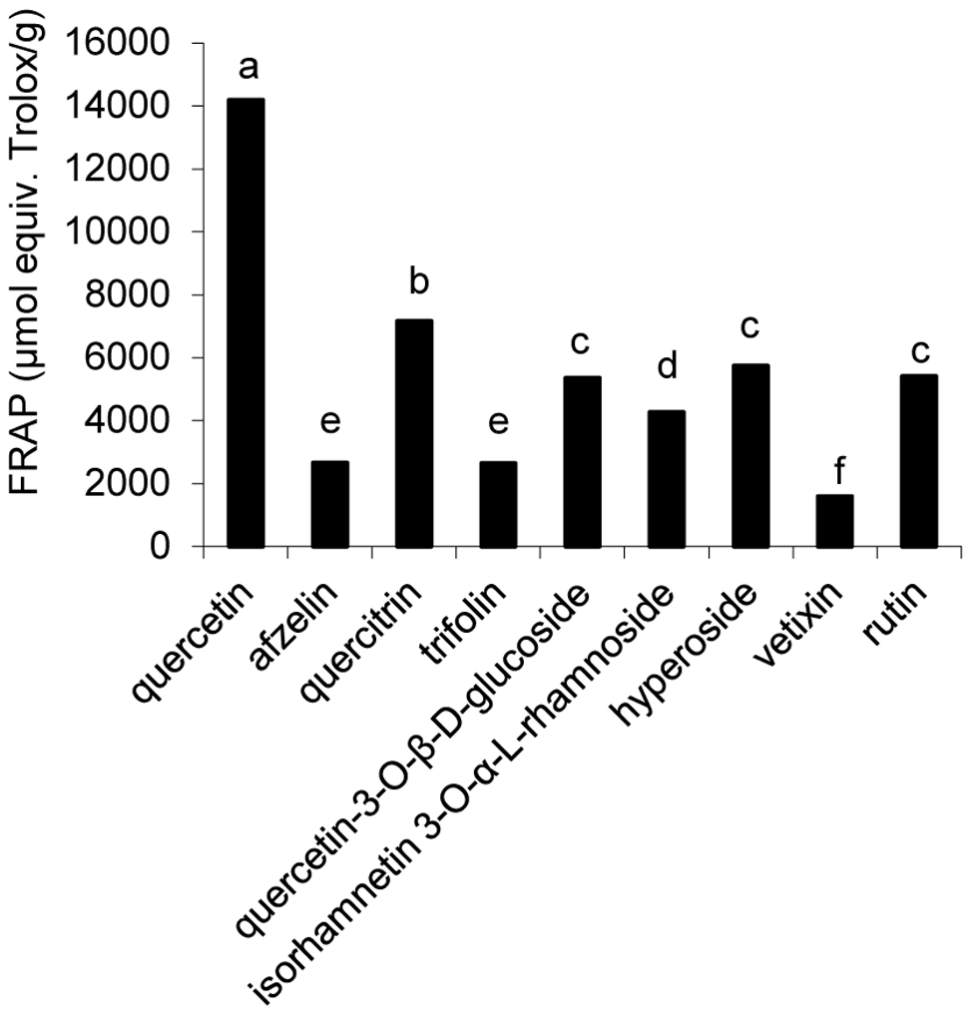
Reducing power of isolated compounds (mean ± SD, N = 3). Bars with no letters in common are significantly different (P<0.05).

In the FRAP assay, quercetin and quercetin glycosides (quercetin, quercitrin, quercetin-3-O-β-D-glucoside, hyperoside and rutin) showed the highest (p<0.05) ferric reducing ability (>5300 µmol equiv Trolox/g) compared with the isorhanmetin glycoside (**6**), kaempferol glycosides (**2** and **4**), or C-glycoside flavonol (**8**) (<4300 µmol equiv. Trolox/g) (Figure 4).

### Lipid peroxidation inhibition of isolated compounds

**Figure 5:**
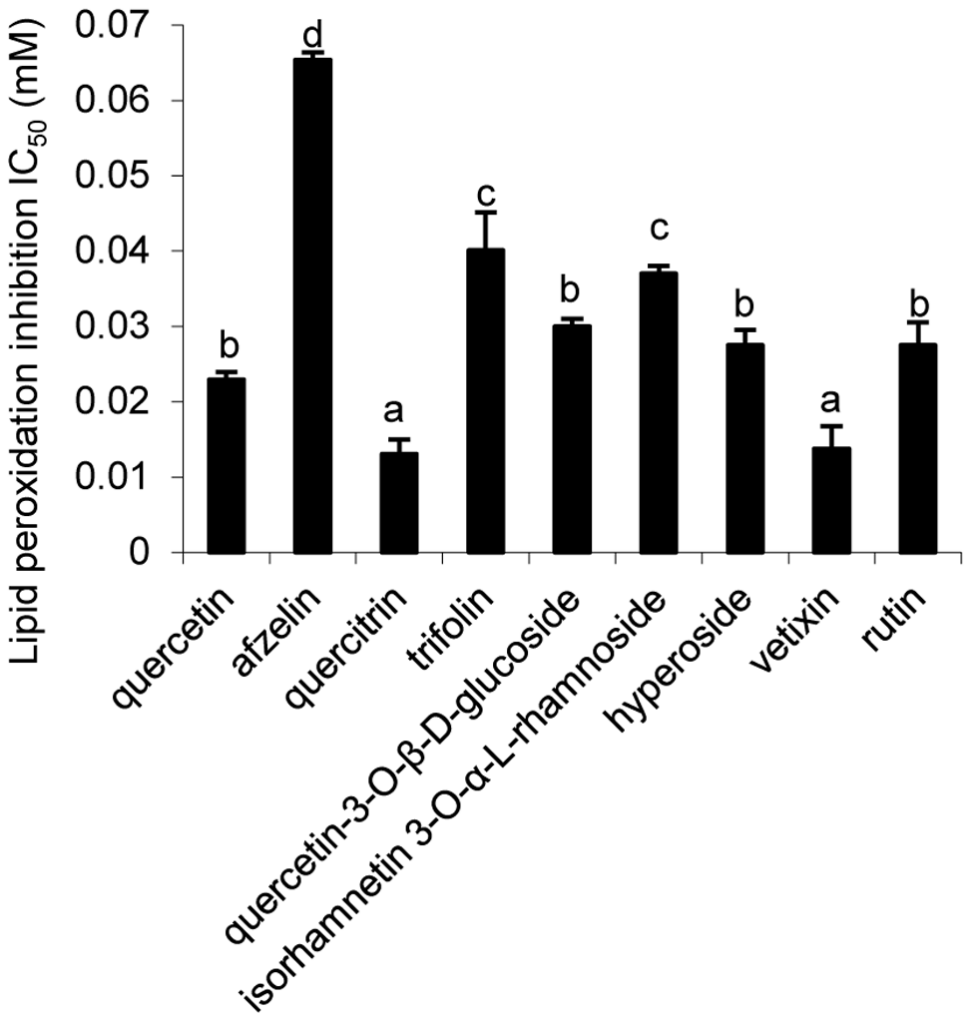
IC_50_ values of isolated compounds for lipid peroxidation inhibition activity (mean ± SD, N = 3). Bars with no letters in common are significantly different (P<0.05).

Vitexin (**8**) and quercitrin (**3**) was observed to have the highest lipid peroxidation inhibitory capacity with the lowest IC_50_ values of 0.014±0.001 and 0.013±0.005 mM, respectively (p<0.05), compared with kaempferol glycosides (0.065±0.003 mM for afzelin and 0.040±0.001 mM for trifolin, p<0.05). Quercetin and other quercetin glycosides, as well as isorhamnetin glycosides, were also found to possess potent lipid peroxidation inhibitory activity (Figure 5).

### Protective effect of isolated compounds on E. coli under peroxide stress

**Figure 6:**
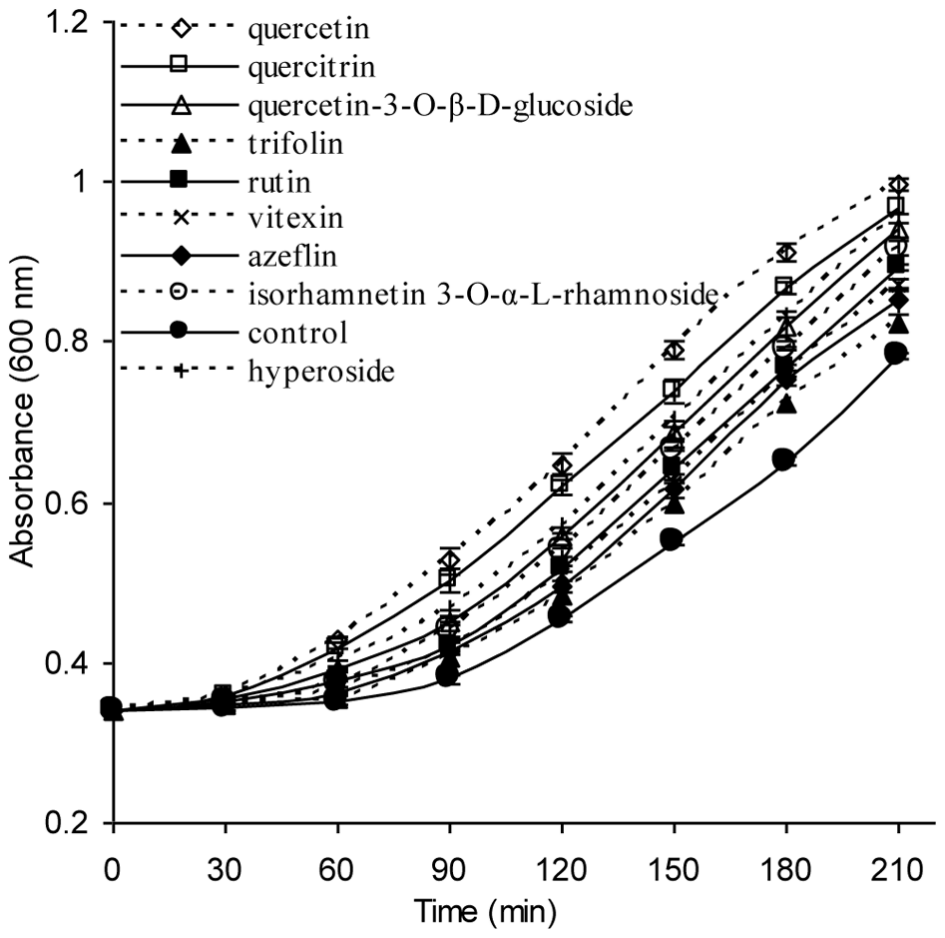
Influence of pretreatment with different isolated compounds on growth in *E. coli* under peroxide stress (mean ± SD, N = 3).

**Figure 7:**
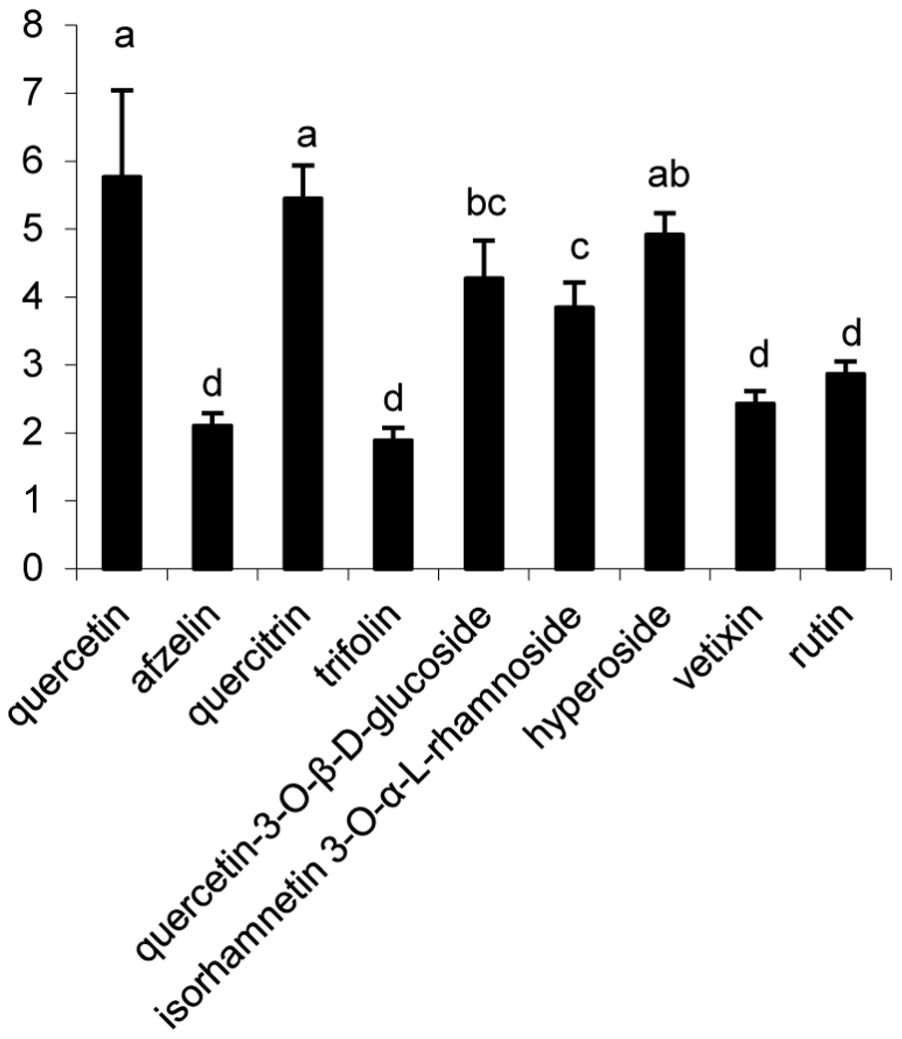
The relative protective effect of isolated compounds on *E. coli* under peroxide stress: t = 30 min, the specific growth rate of *E. coli* in medium containing compounds and 6.5 mM H_2_O_2_/the specific growth rate in medium containing only 6.5 mM H_2_O_2_ (the control) (mean ± SD, N = 3). Bars with no letters in common are significantly different (P<0.05).

In our current research, nine flavonoids were tested on *E. coli* growth under peroxide stress. Addition of 6.5 mM H_2_O_2_ into growing *E. coli* cells (OD_600_ = 0.342) resulted in a 40-min growth arrest (Figure 6). A 90-min pretreatment of *E. coli* by flavonoids **1–9** did not cause considerable effects on duration of the H_2_O_2_-triggered growth arrest. Addition of 6.5 mM H_2_O_2_ into the cells pretreated with flavonoids **1–9** inhibited growth considerably but not completely. In these cultures the earlier recovery of rapid growth was observed. The values of optical density statistically different compared to control were reached in 30–40 min after H_2_O_2_ addition in cultures pretreated with compounds **1–9** (Figure 6). All the isolated compounds exerted a protective effect against the bacterio static action of H_2_O_2_, increasing cell growth rate under peroxide stress to 1.88–5.76 fold compared with that of untreated cells (the control) in 30 min (p<0.05). In particular, the highest activity was revealed in quercetin and quercetin glycosides (quercetin, quercitrin, hyperoside, quercetin-3-O-β-D-glucoside, isorhamnetin 3-O-α-L-rhamnoside and rutin), and their growth rate were 5.76-, 5.45-, 4.91-, 4.27-, 3.84-, and 2.96-fold, respectively (p<0.05), compared with that of untreated cells (the control) 30 min later (Figure 7).

Our results indicated that the antioxidant capacity of the nine compounds varied. Taking into account such parameters as ability of DPPH, ABTS radical scavenging, ferric reducing power, lipid peroxidation inhibition and protection of *E. coli* cells under peroxide stress, the highest antioxidant activities were observed in quercetin and quercetin glycosides.

## Discussion

Owing to the high medicinal values of *Z. bungeanum*, intensive investigations lead to isolation of many classes of secondary metabolites such as flavonoids, alkaloids, amides, lignans, coumarins [8]–[11]. Till now, thirteen flavonoids (two new flavonol glucoside, quercetin 3′,4′-dimethyl ether 7-glucoside and tamarixetin 3,7-bis-glucoside, together with hyperoside, quercetin, quercitrin, foeniculin, isorhamnetin 7-glucoside, rutin, 3,5,6-trihydroxy-7,4′-dimethoxy flavone, arbutin, sitosterol β-glucoside, L-sesamin and palmitic acid) were isolated from the pericarps of *Z. bungeanum* [9]. Studies on individual polyphenols in the leaves of *Z. bungeanum* have not been reported yet, especially the chemical structure of each polyphenol. In this study, nine compounds were isolated and identified from the leaves of *Zanthoxylum bungeanum*, namely quercetin (**1**), afzelin (**2**), quercitrin (**3**), trifolin (**4**), quercetin-3-O-β-D-glucoside (**5**), isorhamnetin 3-O-α-L-rhamnoside (**6**), hyperoside (**7**), vitexin (**8**) and rutin (**9**). The nine isolated compounds are mainly quercetin and kaempferol glycosides, apart from one isorhamnetin glycoside and one c-glycoside flavonal. They are all part of the flavonol glycosides, a class of flavonoids. All the compounds were isolated for the first time in the leaves of *Z. bungeanum*. This is the first report of nine flavonoids isolated from *Z. bungeanum* leaves. Compounds **2, 4, 5, 6** and **8** were found for the first time in the genus *Zanthoxylum*. In literatures, rutin, hyperoside and quercetin were reported to have antioxidant, chelation, anti-carcinogenic, cardio protective, bacterio static, and secretory properties [40], [45]. Afzelin (kaempferol 3-O-α-L-rhamnoside) is a competitive inhibitor of intestinal SGLT1 cotransporter, and it is very effective in reduction of glucose intestinal absorption [35]. Quercitrin (quercetin 3-O-α-L-rhamnoside) is a well-known flavonoid with anti-diarrhoeic activity, sedative activity, anti-inflammatory effect and antifungal activity [32], [46]. Vitexin, a bioactive C-8 glycosylated flavonoid, has been reported to have antiviral, antimicrobial, antioxidant and radioprotection activities [37]–[38]. The various biological activities of these compounds indicated that the leaves of *Z. bungeanum* have health benefit when consumed. These also could explain its frequent addition to the Chinese diet for promoting human health and for disease prevention.

With more than 5000 species known, flavonoids are among the most potent natural antioxidants in plants, and many of them are stronger reducing agents on a molar basis than ascorbic acid [39]–[40]. Their chemical structures consist of a backbone with two benzene rings linked by a pyran chain (C_6_-C_3_-C_6_). The number and position of hydroxyl groups on the aromatic rings determine the polyphenol's antioxidant capacity. Matsuda and others (2002) found that flavones and their derivatives were found to be the most effective antioxidants during a comparative study among different flavonoid structures [41]. In the present study, due to the results of several *in vitro* and *in vivo* antioxidant activity tests, all the isolated flavonoids exhibited potential antioxidant activity. As shown in Figure 2–7, the antioxidant capacities of the nine compounds varied. Taking into account such parameters as ability of DPPH, ABTS radical scavenging, ferric reducing power, lipid peroxidation inhibition and protection effect on *E. coli* cells under peroxide stress, the highest antioxidant activity among the isolated compounds were found in quercetin and quercetin glycosides. Furthermore, quercetin (**1**) had the highest antioxidant activity of all. When the -OH at 3 position on the C ring was glycosylated, its antioxidant activity decreased. The effects of quercetin were compared to those of Trolox. The latter compounds exert its antioxidant effects *in vivo* indirectly by free iron chelating and decreasing production of hydroxyl radicals in the Fenton reaction [28].

The five methods used for determine the antioxidant capacity of the nine flavonoids have complementary effects. All the methods are well designed for determination of the antioxidant activity of pure, isolated compounds, though the results varied in the different antioxidant capacity tests. Dubeau (2010) and Bourassa (2013) pointed out the discrepancies concerning the effect of milk on antioxidant capacity of tea polyphenols might be due to the different methods, more specifically on the phase in which the redox reaction occur [42]–[43]. Due to the different structures of various flavonoids, they will certainly exhibit different antioxidant capacity when the reactions occur in solution or in oil-in-water emulsion. For example, quercitrin (**3**) and vitexin (**8**) displayed higher efficiency to retard lipid peroxidation compared with quercetin (**1**), owing to their chemical structure facilitating their partitioning at the lipid-water interface of the egg yolk micelles [42]. Efficiency of an antioxidant is determined not only by its susceptibility to donate an electron or a hydrogen atom to an oxidant [40] but also by its accessibility to the oxidant [44]. However, quercetin (**1**) displayed higher DPPH, ABTS radical scavenging abilities and better reducing power than quercetin glycosides. Rutin (**9**) displayed less antioxidant activity compared with other quercetin glycosides. This could be attributed to the fact that those larger polyphenols diffuse less readily in aqueous media reducing the overall free radical scavenging ability [42]. Freitas and coworkers proved that the binging affinity of polyphenols increases with the molecular weight and the number of hydrophilic hydroxyl groups. This binding can affect the electron donation capacity of the flavonoids by reducing the number of hydroxyl groups available for oxidation in the media [45]. In *in vivo* test, quercetin and flavonol quercetin, capable of increasing katG expression and catalase activity in *E. coli* and chelating intracellular redox-active iron, showed the most effective protection of bacterial growth after H_2_O_2_ treatment [46]–[52]. Structure-activity relationships revealed that several structural requirements for antioxidants were proposed; the -OH in 4′ position on the B ring and the -OH in 7 position on the A ring possessed high antioxidant activity. The free 7-OH group on the A ring is the main structural feature for the effectiveness of free radical scavenging and protective effect on cells under oxidation. In addition, 2-phenyl substitution due to its aromatic and lipophilic nature, as well as its specific spatial conformation, was found to be effective for the scavenging of free radicals. Finally, the 4′- OH group seems to play an important role for exhibiting the antioxidant activity of these compounds [53]–[56]. These explained the whole powerful *in vitro* and *in vivo* antioxidant activity of compounds isolated from *Z. bungeanum* leaves. B ring and/or A ring with adjacent OH groups could greatly increase their antioxidant ability. The rhamnosyl moiety is present in kaempferol 3-O-α-L-rhamnoside (**2**), isorhamnetin 3-O-α-L-rhamnoside (**6**) and quercetin 3-O-α-L-rhamnoside (**3**), but compound **3** has higher antioxidant activity, indicating that the hexose is not a determinant for their biological activity. The presence of an -OH (**3**) instead of a -H (**2**) or -OCH_3_ (**6**) in the 3′ position of the B ring is a determinant for the potent antioxidant activity. The same tendency of antioxidant activity was also found in kaempferol 3-O-galactoside (trifolin, **4**) and quercetin 3-O-β-D-galactoside (hyperoside, **7**) in which a galactosyl moiety is present in both **4** and **7**, proving that the -OH in the 3′ position of the B ring is a determinant for the potent antioxidant activity. These also explain that quercetin and quercetin glycosides (compounds **3, 5, 7, 9**) have better antioxidant activity compared with kaempferol glycosides or vitexin.

## Conclusions

In our current study, nine flavonoids were isolated for the first time from the leaves of *Z. bungeanum*. Five compounds (**2, 4, 5, 6** and **8**) were found for the first time in the genus *Zanthoxylum*. To learn the mechanisms underlying its health benefits, we further investigated the antioxidant activities of nine isolated compounds using several *in vitro* (DPPH, ABTS, FRAP and lipid peroxidation inhibition assays) and *in vivo* (protective effect on *Escherichia coli* under peroxide stress) methods. Among them, quercetin and quercetin glycosides showed the highest antioxidant activity. Structure-activity relationships indicated that, the -OH in 4′ position on the B ring and the -OH in 7 position on the A ring possessed high antioxidant activity; B ring and/or A ring with adjacent -OH groups could greatly increase their antioxidant ability. Also, due to the different structures of various flavonoids, they will certainly exhibit different antioxidant capacity when the reactions occur in solution or in oil-in-water emulsion. These findings suggest that *Z. bungeanum* leaves may have health benefits when consumed. It could become useful supplements for pharmaceutical products and functional food ingredients in both nutraceutical and food industries as a potential source of natural antioxidants. This study offers a theoretical basis for the further study on the bioactive compounds from *Z. bungeanum* leaves.
